# The Colonial Nursing Association

**Published:** 1899-07-29

**Authors:** 


### THE COLONIAL NURSING ASSOCIATION.

### MR. CHAMBERLAIN'S SPEECH.

At the annual meeting of the Colonial Nursing Association on the 25th inst., the following important speech was made by Mr. Chamberlain: "I have been asked to move, 'That this meeting approves and confirms the annual report of the Colonial Nursing Association and expresses its cordial appreciation of the manner in which the executive committee have given effect to the objects of the Association; and this meeting pledges itself to give its earnest support to the executive committee in their endeavour to raise the funds of the Association to £5,000.' The chairman has explained very fully, though in his modesty he says inadequately, the objects of this Association, and on that head he has really left me very little to add. These objects may be expressed in a sentence—they are to provide for our sick fellow-countrymen abroad the same kindly and skilful treatment which all in sickness have at home. And to fulfil that object the Association undertakes the provision of nurses, makes all the arrangements, pays the passages, organises the whole system, gives information, and invites, in effect, a demand upon its time and attention, and, in addition, so far as its limited funds allow, it is prepared, in those districts which either from their poverty or small population are unable to provide the whole of the expense of the nursing for themselves, to contribute something adequate to the purpose. This provision has undoubtedly had most admirable results. I see from the report that up to the present time something like 40 nurses have been sent abroad, and the reports in all cases are favourable. The demand is increasing, and nothing but want of funds will prevent this Association from completing its work and establishing a similar system in every colony and dependency of the Queen. I am here in my official capacity to testify, as I do most heartily, to the advantages which have already been derived from the labours of this Association, and to the opportunities which are still open to it; and I may say that the Colonial Office is thoroughly satisfied with the experiment which has been tried. We are willing, to the very utmost of our power, to give the co-operation which is needed, and in every way to support the work of this Association. Especially I attach importance to the recommendation which the committee have made, which they speak of as being an ideal—although, like most of their ideals, I think it is extremely practical—that is, that every hospital within our colonies or dependencies which is either supported by the society or by Government funds, should have one or more of these nurses attached to it, who should devote themselves, where circumstances require it, to the service of private patients, and should give the rest of their time to the work of the hospital, which should become their head-quarters. That is an ideal which I hope we may realise before very long, and, as I have said, the results of the experiment are thoroughly satisfactory. Almost every day we receive testimonials in favour of the nurses who have been sent out bearing on the importance and value of their work. Only the other day I saw a letter from Colonel Willcocks, who is in command of the West African Frontier Forces on the Niger, in a particularly unhealthy district, in which he paid a most flattering tribute to the virtues of the ladies who had undertaken service with the forces he commands. He declared his belief that the recovery of some of the young officers who had been struck down by that most deadly enemy of the white man, the black-water fever, was due to the attention and care of the nurses who had been sent out by this Association. But I am sure it is not necessary for me to quote testimonials in favour of the value of a work like this, which must commend itself to every responsible and intelligent person. Nobody can doubt the advantage of skilled nursing in the case of serious illness. Here at home we are endeavouring to secure it in every town and in every village in the country in connection with the District Nurses' Association, and it is universally recognised that the work of the doctor, the surgeon, and the physician is immensely assisted by skilled and sympathetic nursing. But, if it be necessary at home, how much more is it desirable and essential in those tropical colonies where disease is more prevalent, where it is more serious, where the conditions of sickness are more depressing, and where hitherto our sick fellow-countrymen have been left almost entirely to the tender mercies of dirty and indifferent and ignorant natives. Therefore, you will see the field for the operations of such an association is a very large one. The harvest is white, and in this case the labourers are many, for it is, I think, a testimony to the courage and devotion of British women that we find any number of them are willing, and even eager, to take posts, even in the climates reputed to be most unhealthy, and that the applications for such positions are as numerous as those for countries more favourably situated. All that is wanted is a very modest addition to the funds. As Lord Loch has pointed out, the Association is practically self-supporting. Those who enjoy the benefit of its care and attention are expected, as far as their means will allow, to pay for it, but something like a guarantee fund is required in order that the Association may go forth and continue and complete their work. I cannot doubt that their mission will appeal to everybody who has friends and relations in those distant countries—as, indeed, who has not in the present day? It is a mission to all those who are interested in the development of the great Empire of which we form a part. The importance and the value of the Empire is, perhaps, more justly appreciated now than it ever has been—or, at all events, than it has been in recent times—and with that increased appreciation has come, I am glad to think, a deeper sense of the duties, of the obligations, of the responsibilities which come with it. There has devolved upon this country, partly it may be by the special efforts of some of its citizens, partly by circumstances which appear to be fortuitous, partly on the principle, as I hold, of the process of the natural selection of the fittest—there has devolved upon this country a large share of the control of the tropics, of the administration of vast territories and enormous populations, who will owe peace and security to the influence of Great Britain, which has undertaken the administration of these territories, which is founded, I believe, upon justice and upon the sincere and unselfish desire to secure the progress and advancement of the numberless millions who are committed to our charge. If we are pursuing the idea, as I believe we are, in an unselfish spirit, I am perfectly aware also that in its consequences it is of the greatest importance to us, and affects materially our future prosperity and position. Our trade, upon which the very existence of the country depends, tends more and more in the nature of things to become an interchange of products between dissimilar countries, between the temperate countries and the tropical countries, rather than between two or more temperate countries, where the natural advantages and products are the same. Accordingly, we have to consider not merely our duty to our race, but our interests, and I say that our success in the work that has been cast upon us, whether in the matter of government or in the matter of trade, whether in administration or in the development of the Empire, depends mainly upon our power continuously to pour into those regions a supply of suitable agents—men, that is, of character, men of capacity, men of an adventurous spirit, who are ready to risk their lives in the cause of civilisation, in the service of the State to which they belong. Fortunately for us, there is no dearth of such men among us. The demand is great, but the supply is always greater, and I think the most confirmed pessimist will find it difficult to despair of his country as long as he sees that these great qualities of courage, of resolution, of resource, which have distinguished the race in time past and have enabled us to build up this Empire, are still supereminently present in the men, the explorers and the soldiers, the civil officials, the missionaries, and the traders who carry into those regions the great Pax Britannica, and substitute its blessings for the disturbances and the evils which have been created by barbarous customs of intertribal strife and the cruellest forms of slavery. The lives of these men are one continual struggle, they have to fight more with nature than they have to fight with man, and their deeds, although they may not resound with the publicity which attends great feats of arms in the field, are none the less heroic. Many epics might be written, aye, are written in the records of the Colonial Office, of these modern Ulysses who have encountered greater dangers and have overcome greater obstacles than ever were encountered by their classical prototype. I have desired in these few remarks to bring you to the conclusion I desired to press upon you, that is, that lives such as these, so precious to the Empire, the lives of those who are the successors of those who gained the Empire for us, such lives ought not to be wasted. We owe it to them and to ourselves to do all in our power to preserve them, and to see, so far at all events as that may be possible, that when they are struck down, as unfortunately they are often in the course of their duty, by illness, at all events they shall not want the tending of skilful and kindly hands, and that sympathy, that womanly attention, which will be found to be the best anodyne for their pain and perhaps the most effective cure for their disease. I commend most heartily the work of this association."

